# Individual quality of life and the environment – towards a concept of livable areas for persons with disabilities in Poland

**DOI:** 10.1186/s12889-021-10797-7

**Published:** 2021-04-17

**Authors:** Izabela Grabowska, Radosław Antczak, Jan Zwierzchowski, Tomasz Panek

**Affiliations:** SGH Warsaw School of Economics, Institute of Statistics and Demography, Warsaw, Poland

**Keywords:** Quality of life, Disability, Livability, MIMIC, Capability approach

## Abstract

**Background:**

The United Nations Convention on the Rights of Persons with Disabilities [1] highlights the need to create proper socioeconomic and political conditions for persons with disabilities, with a special focus on their immediate living conditions. According to the Convention, these conditions should be built to ensure that persons with disabilities have the potential to enjoy a high quality of life (QoL), and this principle is reflected in the notion of livable areas. The crucial aspect of this framework is the relationship between the individual QoL and the environment, broadly understood as the socioeconomic as well as the technical conditions in which persons with disabilities function.

**Methods:**

The basic research problem was to assess the relationship between individual QoL for the population with disabilities as a dependent variable and livability indicators as independent variables, controlling for individual characteristics. The study used a dataset from the EU-SILC (European Union Statistics on Income and Living Conditions) survey carried out in 2015 in Poland. The research concept involved several steps. First, we created a variable measuring the QoL for the entire population with disabilities. To measure the multidimensional QoL, we used Sen’s capability approach as a general concept, which was operationalized by the MIMIC (multiple indicators multiple causes) model. In the second step, we identified the livability indicators available in the official statistics, and merged them with survey data. Finally, in the last step, we ran the regression analysis. We also checked the data for the nested structure.

**Results:**

We confirmed that the general environmental conditions, focused on creating livable areas, played a significant role in shaping the QoL of persons with disabilities; i.e., we found that the higher the level of the local Human Development Index, the higher the quality of life of the individuals living in this area. This relationship held even after controlling for the demographic characteristics of the respondents. Moreover, we found that in addition to the general environmental conditions, the conditions created especially for persons with disabilities (i.e., services for this group and support for their living conditions) affected the QoL of these individuals.

**Conclusions:**

The results illustrate the need to strengthen policies aimed at promoting the QoL of persons with disabilities by creating access to community assets and services that can contribute to improving the life chances of this population.

**Supplementary Information:**

The online version contains supplementary material available at 10.1186/s12889-021-10797-7.

## Background

The United Nations Convention on the Rights of Persons with Disabilities (UNCRPD) set several guidelines that have led to the adoption of a new approach to formulating public policy aimed at the population with disabilities. The new approach is based on the right of persons with disabilities to enjoy a high quality of life without discrimination or exclusion [1]. The UNCRPD focuses on the macro-level systems that are expected to create proper socioeconomic and political conditions for persons with disabilities, with a special focus on their immediate living conditions. These conditions should be built to ensure that persons with disabilities have the potential to enjoy a high quality of life (QoL). The notion of QoL reflects subjective and objective assessments of people’s living conditions at the individual level. Hence, QoL can be seen as a link between the general values and rights embodied in the UNCRPD in particular, and the personal life of the individual [2–5]. The crucial aspect of this framework is the relationship between the individual QoL and the environment, understood as the socioeconomic as well as the technical conditions in which persons with disabilities function [6]. According to the UNCRPD, the interactions between persons with disabilities and the environment create a degree of inclusion and participation in all life spheres for this group.

Therefore, our aim in this article is to examine the relationship between the multidimensional quality of life of persons with disabilities and the level of livability, as expressed by the environmental conditions at the local level in Poland [7–9].

The article has several sections. In the first section, we describe our conceptual framework, the theoretical background, and our research design. In the subsequent sections, we present three main research steps: the conceptual part, the measurement part, and the analytical part. In the next section, we present our final results, and we conclude with a discussion of the limitations of our analysis and our conclusions.

### Research design and data

Our basic research design consists of an assessment of the relationship between the individual QoL of the population with disabilities as a dependent variable, and livability indicators as independent variables, controlling for individual characteristics. Our theoretical considerations are based on two crucial concepts: QoL and livability (conceptual framework). Based on the literature review, we establish the measurement model for the QoL of the entire population with disabilities. We then measure the livability levels by identifying the livability indicators and preparing them statistically for use in the regression model (measurement framework). Finally, in the last step, we run the regression model of QoL against livability indicators, controlling for the socioeconomic characteristics of the respondents (analytical framework).

**Fig. 1:**
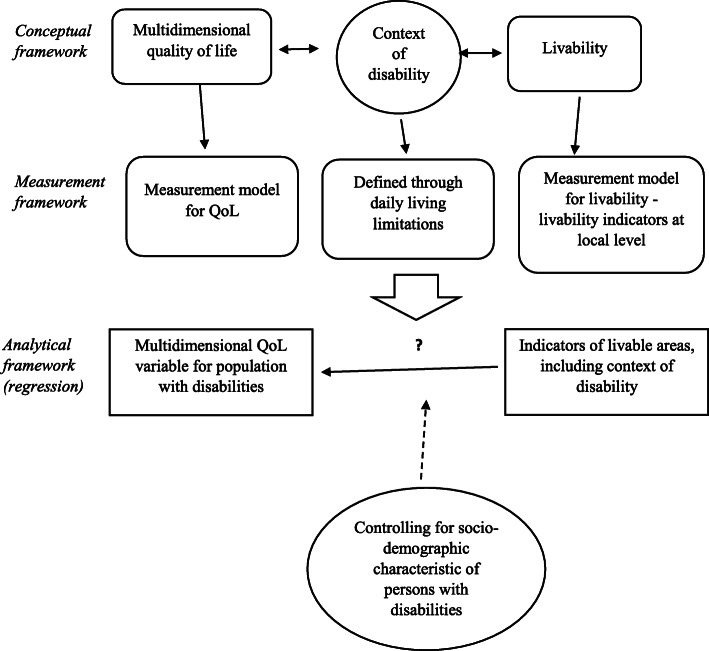
Research design. Source: own study

The whole research design, which is composed of three steps (conceptual, measurement, and analytical parts), is presented in Fig. 1.

In our research, we use three different data sources. Data on individual quality of life are drawn from the European Survey on Income and Living Conditions (EU-SILC), conducted in 2015 in Poland [10]. This survey was carried out under an EU resolution on a representative sample of the Polish population aged 15 or older, and the statistical unit was a person living in a private household. The total sample size for 2015 was 27,997, and the sample size of persons with disabilities was 6615. This latter sample is used for further analysis. Of this population, 58.3% were women, 49.9% were aged 65 or older, 68.2% had less than secondary education, and 80.7% were not working. The livability indicators at the local level are drawn from data collected for 2015 in the local database of the Central Statistical Office. The local development indicators at the local level come from the Local Human Development Index (LHDI) calculated for Poland for 2010 and 2007 [11].

### Conceptual framework

The conceptual framework deals with two basic concepts within the context of disability. The first concept is the quality of life, with a special focus on the multidimensional character of and the measurement dilemmas associated with this indicator. The second concept refers to livability and the creation of the living environment at the local level.

Ideas about what constitutes “the good life” are rooted in ancient philosophy. Based on these foundations, three main constructs are used in the analysis of quality of life and well-being: hedonic well-being, eudaimonia, and life satisfaction. Hedonic well-being underlines the importance of emotions, affect, and subjectivity; eudaimonia points to the value of self-development and self-realization; and life satisfaction refers to cognitive aspects of well-being [12].

Quality of life as a general concept has been studied in many fields, including economics, political science, psychology, philosophy, and medical science. The concept of QoL was introduced into the public discourse in the 1960s as an alternative to the prevailing focus on social development goals, which were at that time defined as improvements in material living conditions [13]. Although the term is commonly used, there is no single, universally accepted definition of quality of life [14]. The World Health Organization’s definition focuses on individuals’ perceptions of their position in life, and the extent to which it corresponds with their expectations. Other definitions include the satisfaction of needs, objective and subjective evaluations of different dimensions of life, agency, and the meaning of life. Interest in measuring QoL is increasing in the area of health care, where it is identified as an outcome of the efficacy of the treatment [15]. Hence, as the concept of QoL is multifaceted, multidimensional, and ambiguous, a clear definition of it is needed before beginning research on quality of life [16]. For the purposes of this article, we applied the individual-referenced definition outlined by Schalock et al. [4], in which they described QoL as a multidimensional phenomenon composed of core domains influenced by personal characteristics and environmental factors. The authors argued that these core domains are the same for all people, although their relative value and importance may vary individually.

Alongside these various definitions, different tools for measuring this phenomenon have been developed e.g., [1, 16–19]. Generally, there are two approaches to measuring QoL among persons with disabilities:

1. a measure constructed for the whole population and used to assess the QoL of different sub-groups, including persons with disabilities; and
2. a measure constructed and used specifically to assess the QoL of persons with particular limitations or disabilities.

The most complex measurement guidelines based on the first approach are provided by the final report of the Sponsorship Group on Measuring Progress, Well-being and Sustainable Development and the Task Force on the Multidimensional Measurement of Quality of Life [20,21], which was accepted by the European Statistical System Committee. This proposal represents an extension of the QoL measurement concept of Berger-Schmitt and Noll [22] operationalised in the framework of the European System of Social Indicators, which refers to recommendations of the Report on Measurement of Economic Performance and Social Progress [23]. Those reports stressed the multidimensional character of QoL, as well as the importance of combining both subjective and objective measures of QoL. Moreover, it was clearly stated that QoL should be assessed at both the individual and the community levels. In its final report, the Task Force on the Multidimensional Measurement of Quality of Life identified nine dimensions to be measured within the framework of the European Statistical System [20]. Each dimension comprises a set of indicators of a subjective and an objective character. This system of indicators enables the analysis of different life aspects within each dimension and their changes over time, as well as the relative assessment of QoL of individuals or households. However, it does not provide an explicitly formulated guide for operationalising the measurement, or a synthetic measure of QoL.

In the second approach, the concept of quality of life is used to assess the personal outcomes for persons with disabilities guaranteed under the UNCRPD [24]. In this approach, quality of life is also considered as a multidimensional construct that includes physical, mental, and social dimensions [19], but it is used in the context of a particular disability or impairment. The influence of a particular type of disability or affliction on a person’s quality of life is visible, and can be measured in different domains using both subjective and objective measures i.e. [25, 26].

These two approaches (general quality of life and QoL developed for persons with limitations) have important similarities. Both approaches assume that QoL should be composed of the same factors and relationships for all people; is experienced when a person’s needs are met and when the individual has the opportunity to pursue life enrichment in major life activity settings; is comprised of both subjective and objective components; and is a multidimensional construct influenced by both individual and environmental factors [5, 24]. However, in the overall quality of life measurement approach, which measures QoL in the total population, the scope of the dimensions considered is relatively broad; whereas in the approach for measuring the QoL of persons with disability, the starting point is assessing functioning connected with various limitations.

An interesting proposal for measuring QoL that combines elements of both of the above-mentioned approaches is the capability approach, which was developed and refined by Sen [27–33]. This approach can be used to measure the QoL of the entire population, and specifically of the population with disabilities. The capability approach has been synthesised and practically applied by numerous authors in a wide variety of fields [34–41]. This concept is based on the assumption that commodities themselves are not crucial to achieving a high quality of life. Instead, Sen argued, it is the properties of these commodities that enable individuals to achieve their desired lifestyles. The term “capabilities” refers to a person’s effective possibilities of realizing achievements and fulfilling expectations; whereas the term “functionings” refers to a person’s “beings and doings” that lead to these realized achievements and fulfilled expectations [42]. To transform commodities into capabilities, three sets of conversion factors – personal, social, and environmental – are needed [36, 43]. Personal conversion factors (personal characteristics, such as metabolism, physical condition, intelligence, or gender) influence the types and degrees of capabilities a person can generate from commodities. Social conversion factors come from the society in which one lives (characteristics of social settings, social institutions, and power structures, such as social norms, public policies, societal hierarchies, rule of law, political rights, etc.). Environmental conversion factors emerge from the physical or built environment in which a person lives (environmental characteristics, such as climate, infrastructure, institutions, and public goods). The achieved functionings are the result of personal choices selected from the capabilities available, and are subject to personal preferences, social pressures, and other decision-making mechanisms. Moreover, they are constrained by personal, social, and environmental characteristics [36, 44]. In the context of inequality analysis, people must have equal opportunities to function in their preferred way [27], as only then are they free to determine their capabilities – i.e., their potential, or possible ways of functioning – and to maximize their quality of life in accordance with these capabilities.

In this article, we have chosen to use the capability approach as a conceptual basis for the measurement model of the QoL. Moreover, we have constructed a measurement model for the whole population that we then apply to the population with disabilities. This macro-level approach could be used to create public policy guidelines aimed at the population with disabilities.

Living conditions, which should be shaped by public policy, especially at the local level, are associated with the concept of livability. It is rarely used in disability research, even though environmental conditions (such as accessible space) are especially important for this group of people. The concept of livability originally comes from the literature on public management. It was used for the first time in the U.S. in the 1970s in the context of discussions about urban sprawl and the problems caused by the degradation of the natural environment [45]. Since then, the concept has mainly been developed in urban studies, although it originally also referred to rural areas [7, 46]. While there are many definitions of livability, the term is often used in connection with concepts such as quality of life, living environment, the quality of the place of residence, and sustainable living [8, 9, 47].

The term “livability” was originally used to refer to residents’ satisfaction with the area they are living in, and especially with the perception of living conditions in a particular area that should be shaped by both residents themselves and local authorities [7]. This approach underlined two important elements of the livability concept: (1) its relationship with the environment in which the local community functions; and (2) the importance of focusing on the short-term perspective. Many authors have characterized the livability concept as a set of elements that make life in a particular locale easier or more comfortable. Among these elements are economic opportunities, public security, medical services, mobility, and recreation [47–49]. These elements come from the social, economic, and technical (psychical) sphere, and they create possibilities to realize individual QoL [50]. The interactions between the conditions created in the environment and the individual life situation determine the level of QoL [51, 52]. In this context, spatial planning plays an important role, especially in cities [53].

Recently, the livability concept has mainly been used in the context of meeting the social needs of residents [54]. Therefore, the aim of creating livable areas is connected with social change, in particular through public policy, which should be initiated in those areas. This understanding of livability is in line with that in the UNCRPD, which also takes as a starting point the rights and needs of one particular group – in this case, persons with disabilities – for whom public authorities should create the environmental conditions needed to enable them to realize their desired QoL. Having access to proper environmental conditions is seen as an essential component of the human rights of persons with disabilities.

An important issue raised in livability studies is the measurement of this phenomenon. Two streams in measurement approaches can be distinguished. The first deals with satisfaction with the area where an individual lives, and its determinants [7, 55, 56]. Howley et al. [7] divided those determinants into two groups: (1) the first group of determinants are connected with livability (access to employment, social, educational and cultural services, security, housing, etc.), (2) while the second group of determinants correspond to individual characteristics (age, sex, family status, etc.). Cheshmehzangi [57] also identified similar factors, which he grouped into economic, social, cultural, and environmental categories. The second stream refers to more objective measures of livability. There are many partial indicators created by researchers to measure the level of livability of particular areas (performance indicators) [56, 58, 59].

## Methods

### Measurement framework

The measurement part refers to the definition of the population with disabilities, measurement models of QoL, and livability. The identification of persons with disabilities was based on limitations in activities of daily living (ADL), a commonly used measure of disability. This question has three categories: 1) strongly limited in daily activities; 2) limited, but not strongly; and 3) not limited at all. All persons who were limited in their activities to some extent (1 and 2) were defined as those with disabilities.

To establish a measure of multidimensional QoL, we used the guidelines of the European Statistical System [20, 21] due to its multidimensionality and potential for operationalization. All information on the access to the EU-SILC dataset can be found on the URL (https://ec.europa.eu/eurostat/web/microdata/european-union-statistics-on-income-and-living-conditions) – access on May 2020. To conceptualize the measurement approach, we used Sen’s capability approach [60, 61].

Our QoL measurement model was based on the MIMIC model, which was formulated by Hauser and Goldberger [62], and was popularized by Jöreskop and Goldberger [63]. The operationalization of QoL in the MIMIC model, according to Krishnakumar [64], was performed as follows. Capabilities are endogenous latent variables, which can be estimated on the basis of chosen achieved functionings, and are represented by observable exogenous variables (observable symptoms of QoL). The combined analysis of capabilities sets and achieved functionings enables the multidimensional measurement of QoL. Conversion factors (individual, social, and environmental), which are represented by exogenous variables, positively or negatively influence an individual’s capabilities.

The starting point for establishing the MIMIC model to measure QoL was to assign capabilities to the dimensions of quality of life presented in the European Statistical System [20, 21]. The model includes nine dimensions: material living conditions, productive or main activity, health, education, leisure and social interactions, economic and physical safety, governance and basic rights, natural and living environment, and overall experience of life. Each of these dimensions is represented by a set of determinants (including individual characteristics of the respondents, such as gender, age, place of residence, or health status) and a set of symptoms, which are drawn from a directly observable list of variables in the EU-SILC questionnaires. The list of symptoms for each domain is included in Annex 1.

**Fig. 2:**
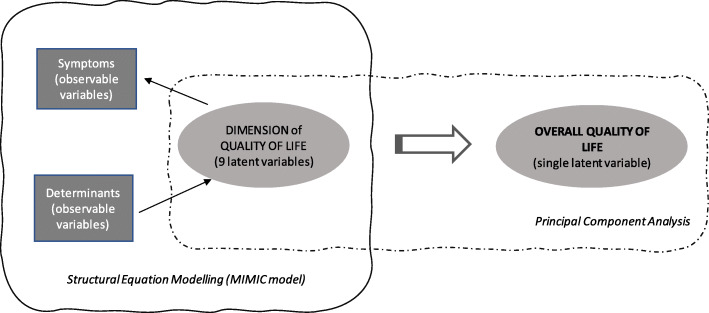
The measurement model of the QoL. Source: own study

Each life domain is represented by an unobservable latent variable, which can be estimated based on two sets of observable variables. First, in the reflective part of the model (symptoms), these variables can be interpreted as realized functionings. The formative part of the model (structural sub-model) is constructed based on the individuals’ personal, social, and environmental exogenous characteristics, which are the conversion factors that strengthen or weaken their capabilities, and influence the process of the transformation of available resources into achieved functioning. To estimate the overall life quality indicator for each person, we used a formative approach [65–67]. The formative indicators included in this approach are considered as determinants of a multidimensional latent variable. In our study, the overall quality of life is described as a latent variable influenced by the dimension (group) quality of life indicators. In this case, the measurement model is based on a principal component method as an aggregation method, which is often used for formative indicators [68]. In this method, it is assumed that the overall quality of life indicator is a linear combination of the dimension (group) life quality indicators, and that there is no measurement error [69]. Finally, the quality of life scores were standardized. The analytical framework is presented in Fig. 2. The results of the MIMIC model estimations (for the entire population – with and without disabilities) are presented in the Annex 2.

Altogether, within the MIMIC procedure we identified 43 indicators (symptoms of achieved functioning that are grouped into nine domains): material conditions: eight symptoms, productivity: eight symptoms, health: two symptoms, education: two symptoms, leisure and social interaction: nine symptoms, economic security and physical safety: four symptoms, government and basic rights: two symptoms, natural and living environment: two symptoms, overall experience of life: four symptoms.

To operationalize the livability level, we have chosen to measure it using a set of appropriate indicators. Livability, as elaborated at the conceptual level of the paper, refers to the living conditions in the local area. This also implies that the measurement should be made at the local level. In the Polish context, we decided to use the LAU level 1 (powiaty). As well as reflecting the local character, an advantage of using the LAU level 1 is that more data (indicators) are available at this level than at the LAU level 2 (gminy). This is especially the case for indicators of public policy aimed at the population with disabilities. Certain indicators that reflect the livability level of a local area in the context of the situation of the population with disabilities can be grouped into two categories:

- those affecting the whole population (with and without disabilities). It should, however, be noted that the same environmental settings may affect the quality of life of the population with disabilities differently than the population without disabilities.
- those that are specifically related to various disabilities [70].

We decided to use simultaneously two groups of indicators based on the above classification.

The first indicator refers to the general environmental conditions created at LAU level 1 for the total population. For this purpose, we used the Local Human Development Index, LHDI [11]. The LHDI refers to the general living conditions in the powiat, and is a very complex measure, although it does not distinguish between the conditions created for the population with and without disabilities. The LHDI consists of three dimensions (health, education, and wealth), and is reflected by several indicators: life expectancy at birth, crude death rates, the fraction of children aged 3–4 taking part in pre-school education, the average results of mathematics and science final exams at the lower-secondary educational level, and the average level of wealth of the inhabitants [11]. The higher the value of the indicator, the higher the general livability level. The LHDI for Poland was calculated for 2007, 2008, 2009, and 2010. In the further analysis, we decided to use the LHDI measured for 2010 as a general approximation of the environmental conditions created for the entire population. There is a time lag between the basic dataset used to establish individual QoL (2015) and the LHDI (2010). However, in the past, this measure has been quite stable over time for Poland.

The second group of indicators refers to the environmental conditions established with a focus on persons with disabilities. In the first step, we searched for proper partial indicators that reflect, at least to some extent, public policies aimed at the population with disabilities at the LAU level 1. We found that while each of proposed partial indicators alone do not provide enough information on the specific conditions for persons with disabilities in the powiats, the proposed group of indicators provide an approximation of such information. The proposed indicators refer to the livability concept in the economic and social spheres. Indicators that refer to the technical sphere, defined as the accessibility of public spaces for persons with different disabilities, are not included in the list of indicators below due to a lack of such data at the LAU level 1 for Poland. The set of partial indicators is as follows:

- Employment activity: the number of unemployed persons with disabilities in relation to the number of persons with disabilities in the powiats. This indicator reflects not only the economic activity of the population with disabilities, but also the accessibility of public employment services.
- Social assistance: the number of families receiving support from social assistance due to disability in relation to the number of persons with disabilities in the powiats. This indicator captures not just cash benefits, but also (as is crucial here) social services such as care services (basic and specialized). For this reason, we treat this indicator as a reflection of the accessibility of basic social services.
- Access to health care: this is measured by the number of general practitioners in relation to the number of persons with disabilities in the powiats. This indicator reflects the general accessibility of basic health services at the powiat level.
- Primary education: this is measured as the number of special units in primary schools and gymnasiums for children and youth with special learning needs. This indicator reflects the accessibility of proper educational services (at the basic level) relatively close to the place of residence.

The above-mentioned indicators come from an online database of the Polish Central Statistical Office. The data are from 2015, as this is the period used for the QoL measurement. We assume that these variables express the attitudes of local authorities towards creating conditions favorable for persons with disabilities in the powiats. The above-mentioned indicators are correlated. Therefore, in order to introduce the full information contained in these indicators into the regression model, we applied the principle component method. The new variables established in that method fulfil the following criteria: (1) they are not corelated (orthogonal); (2) they are normalized (the sum of squares of linear combination coefficients are equal to one); and (3) they appropriately approximate the real variables. We decided to use in the regression model the principle components, which have meaningful interpretations. To do so, we had to rotate the results of the principle component analysis. Finally, we have accepted the model with two components (factors). The first factor is comprised of access to health care and primary education, and is related to the services available for persons with disabilities. The second factor is related to living conditions, and includes two variables: employment activity and social assistance. Those two principal components account for 74.9% of the total variance of the data. Both factors are then used for regression models to assess the influence of environmental factors on overall quality of life.

Indicators from both groups are used in the regression analysis of the multidimensional quality of life against the livability indicators. The indicators reflecting the environmental conditions for persons with disabilities (living conditions and services factors – second group indicators) are orthogonal, but the living conditions variable is highly correlated (r = 0.69) with the LHDI (first group indicator). Hence, we have prepared two separate regression models: one for the LHDI, and another for the environmental conditions for persons with disabilities.

### Analytical framework - regression model

Our basic research design refers to the regression model with the multidimensional QoL score as a dependent variable and indicators that express the livability level as an independent variable, controlling for the socioeconomic characteristics of individuals.

The most commonly used method for modelling geographical data is multilevel modelling. Therefore, in the first step of the analysis, we checked the assumptions for this approach. First, we assessed the nested structure of the data, whereby level 1 consists of individuals and level 2 consists of LAU level 1 (powiats). Next, we ran empty models for the two-level structure. We then calculated the ICC (interclass correlation coefficient), which turned out to be very low (ICC = 0.004), suggesting that a two-level structure is unnecessary. Moreover, we checked the design effect (deff = 1), which also suggests that the data on QoL are not hierarchical at the LAU level 1. As our initial assessment pointed to the inappropriateness of multilevel structure of the data, we decided to apply one- instead of two-level models to answer the research questions. As the dependent variable – i.e., the individual quality of life score – is a continuous variable, we applied an OLS regression with a step-wise procedure. Control variables were gradually introduced into the model.

## Results

### Characteristics of the population with disabilities in Poland

The first step in our analysis was to briefly describe the characteristics of the population with disabilities in Poland based on the data from the EU-SILC survey, as well as to perform a descriptive analysis of the dependent variable (quality of life scores) and the independent variables (livability indicators assigned to each respondent according to their place of residence). The descriptive statistics of those variables are provided in the Table 1.

**Table 1:** Descriptive characteristic of independent variables

	Overall Quality of Life		LHDI_2010		Environment - services		Environment – living conditions		Sample	
	Mean	Std Dev.	Mean	Std Dev.	Mean	Std Dev.	Mean	Std Dev.	n	%
**Total**	0.00	1.00	46.13	15.39	0.00	1.00	0.00	1.00	6615	100.0
**Sex**										
Man	0.08	1.02	45.94	15.69	0.01	1.04	−0.02	0.99	2756	41.7
Woman	−0.06	0.98	46.27	15.16	−0.01	0.97	0.02	1.01	3859	58.3
**Age**										
below 45	0.27	1.12	46.78	14.92	0.02	0.94	−0.03	1.00	849	12.8
45–54	0.01	1.01	43,59	13.46	−0.16	0.79	−0.02	0.97	757	11.4
55–64	−0.07	0.98	46.17	15.58	−0.03	1.00	0.01	0.99	1707	25.8
65–74	0.01	0.98	45.97	15.42	0.01	1.00	0.05	1.02	1581	23.9
75+	−0.12	0.92	47.01	16.21	0.09	1.12	−0.03	1.01	1721	26.0
**Household size**										
single	−0.28	0.99	48.71	17.00	0.21	1.18	−0.06	0.98	1169	17.7
2 persons	0.07	1.00	47.50	15.66	0.07	1.02	0.04	1.02	2572	39.0
3 persons	0.05	1.01	45.76	14.38	−0.06	0.94	−0.06	0.98	1138	17.3
4 persons	0.17	0.98	45.99	15.61	0.01	1.03	0.02	1.03	807	12.2
5 or more persons	0.09	0.94	40.10	10.97	−0.37	0.45	0.05	0.99	906	13.7
**Marital status**										
single	−0.14	1.09	46.73	16.19	0.04	1.03	−0.06	0.95	1067	16.2
married	0.19	0.94	45.40	14.73	−0.06	0.93	0.02	1.01	3919	59.5
divorced	−0.34	1.11	50.56	16.65	0.27	1.14	0.08	1.09	421	6.4
widowed	−0.26	0.96	46.31	15.85	0.04	1.08	−0.04	0.98	1606	24.4
**Place of residence**										
cities 500 k+	0.23	1.00	72.65	16.04	1.85	1.38	−0.52	0.63	681	10.3
cities 100 k–499 k	0.10	1.02	57.03	7.95	0.65	0.94	0.03	1.02	1160	17.6
towns 20 k–99 k	−0.04	0.99	45.70	9.88	−0.30	0.36	0.14	1.09	1239	18.8
towns less than 20 k	0.02	1.05	38.16	8.29	−0.42	0.37	0.05	0.91	954	14.5
rural	−0.11	0.96	37.29	9.84	−0.49	0.33	0.04	1.02	2556	38.8

Source: own calculations (*n* = 6592)

The highest quality of life scores were noted for men, and for people who were below age 45, living in four-person households, married, and from the biggest cities. These groups were found to enjoy the highest quality of life. The most excluded groups (with the lowest QoL) were identified as women, the oldest age groups, single and divorced individuals, and people living in rural areas.

The total average value for the LHDI (first group indicator) was 46.13. Women were found to have above-average scores, regardless of their age and marital status. We also observed a clear household size and place of residence gradient. The LHDI score decreased with household size, and rose as the size of the place of residence increased. Residents of big cities clearly had the highest LHDI scores.

The values for both environmental variables that reflect the livability levels of persons with disabilities (second group indicators) were standardized. The values for services were highest for the oldest age group, singles, and people living in big cities. The maximum scores for living conditions were observed for people who were aged 65–74, living in five-person households, divorced, and inhabitants of mid-sized towns.

### Model results

Two separate models for the two livability indicators were taken into account in our research: the LHDI, as an approximation of general living conditions (first group indicator), and two variables that reflect the environmental conditions for people with disabilities (second group indicator). The control variables reflect the typical characteristics of individuals: sex, age, household size, marital status, and type of place of residence. We excluded variables such as education or income because they were used to create the dependent variable (QoL). We developed four models for each of the independent variables, separately for the LHDI and for the environmental conditions for people with disabilities, and introduced the control variables sequentially. Thus, in Tables 2 and 3, we present the results of eight models.

**Table 2:** The relationship between local HDI and individual quality of life – results of OLS regression

	Model 1			Model 2			Model 3			Model 4		
Variable	Coef.		Std. Err.	Coef.		Std. Err.	Coef.		Std. Err.	Coef.		Std. Err.
**lhdi_2010**	0.009	***	0.001	0.009	***	0.001	0.009	***	0.001	0.007	***	0.001
Sex (ref. man)												
woman	−0.012	***	0.023	−0.084	***	0.023	−0.039		0.024	−0.041	*	0.024
Age (ref. below 45)												
45–54	−0.007		0.046	−0.065		0.046	−0.231	***	0.047	−0.225	***	0.047
55–64	− 0.016	***	0.038	−0.145	***	0.040	−0.341	***	0.043	−0.333	***	0.043
65–74	−0.009	**	0.039	−0.055		0.041	−0.257	***	0.045	−0.252	***	0.045
75+	−0.024	***	0.039	−0.167	***	0.041	−0.307	***	0.047	−0.299	***	0.047
Household size (ref. 71 person)												
2 persons				0.298	***	0.033	0.000		0.038	0.006		0.038
3 persons				0.240	***	0.040	−0.023		0.043	−0.007		0.043
4 persons				0.285	***	0.044	−0.010		0.047	0.012		0.047
5 and more persons				0.283	***	0.042	0.003		0.045	0.043		0.046
Marital status (ref. married)												
single							−0.542	***	0.042	−0.537	***	0.042
divorced							−0.522	***	0.052	−0.532	***	0.052
widowed							−0.376	***	0.035	−0.370	***	0.035
Place of residence (ref, 500 k+)												
100 k–499 k										−0.012		0.056
20 k–99 k										−0.017		0.060
less than 20 k										0.000		0.065
rural										−0.132	**	0.062
_cons	−0.023		0.049	−0.536		0.062	0.024		0.070	0.150		0.108

Source: own calculations

**Table 3:** The relationship between environmental factors and individual quality of life – results of OLS regression

	M1			M2			M3			M4		
**Variable**	**Coef.**		**Std Err.**	**Coef.**		**Std Err.**	**Coef.**		**Std Err.**	**Coef.**		**Std Err.**
**Services**	0.088	***	0.016	0.097	***	0.017	0.097	***	0.016	0.030		0.024
**Living Conditions**	−0.040	***	0.013	−0.043	***	0.013	−0.045	***	0.013	−0.036	***	0.013
Sex (ref. man)												
woman	−0.162	***	0.028	−0.118	***	0.028	−0.072	**	0.028	−0.079	***	0.028
Age (ref. below 45)												
45–54	−0.196	***	0.056	−0.185	***	0.056	−0.294	***	0.057	−0.282	***	0.057
55–64	−0.276	***	0.047	−0.255	***	0.049	−0.385	***	0.052	−0.367	***	0.052
65–74	− 0.202	***	0.048	− 0.153	***	0.051	−0.289	***	0.054	−0.275	***	0.054
75+	−0.324	***	0.048	−0.224	***	0.052	−0.325	***	0.057	−0.313	***	0.057
Household size (ref. 71 person)												
2 persons				0.346	***	0.036	0.034		0.044	0.042		0.044
3 persons				0.299	***	0.047	0.002		0.052	0.027		0.052
4 persons				0.321	***	0.052	0.009		0.057	0.048		0.057
5 and more persons				0.288	***	0.051	−0.012		0.056	0.058		0.056
Marital status (ref. married)												
single							−0.495	***	0.053	−0.489	***	0.052
divorced							−0.542	***	0.061	−0.562	***	0.060
widowed							−0.390	***	0.042	−0.378	***	0.042
Place of residence (ref, 500 k+)												
100 k–499 k										−0.028		0.068
20 k–99 k										−0.085		0.078
less than 20 k										−0.120		0.081
rural										−0.272	***	0.077
_cons	0.324		0.043	0.000		0.058	0.494		0.069	0.614		0.095

Source: own calculations

The results confirmed that the environmental conditions – both the general (local HDI) conditions and those designed to support persons with disabilities – played a significant role in shaping individual quality of life for persons with disabilities.

In the first set of regressions, the local HDI was found to have a positive influence on individual quality of life in all four models. In the first model, in which we assessed the influence of the local HDI controlled only by the age and gender of the individuals, with one point of increase of HDI each, the quality of life index grew by 0.009 points. The same effect was observed in the second model, which additionally controlled for household size; and in the third model, to which marital status was added. When the place of residence of an individual was added, the observed effect was slightly smaller. However, in all of the models, the influence of the local HDI was found to be significant at the 0.001 level. This result suggests that the higher the level of development of the local area (powiat), the higher the quality of life of individuals with disabilities living in this area.

In the second set of regressions, we examined the influence of the environmental conditions on the quality of life of persons with disabilities. Both variables – services and living conditions – were also found to have significant effects on quality of life. Due to the nature of the environmental variables, the direction of the influence was different: i.e., it was positive for services and negative for living conditions.

In the first model, an increase in the services score by one point was accompanied by an increase in the overall quality of life by 0.088 points. An increase in living conditions deprivation of one point decreased the quality of life by 0.040. Similar results were observed models 2 and 3; i.e., after controlling for household size and marital status. These results suggest that the strength of the impact of the environmental conditions was unchanged even after we accounted for a person’s demographic characteristics.

The only important difference we observed was in model 4 after the inclusion of the place of residence, and then only for one of the environmental factors. Services were no longer found to be significant after the place of residence was introduced into the model. This result means that the availability of services was highly related to the size of the place of residence. Living in a rural area was shown to have a negative influence on the individual quality of life, as it was found to be associated with limited access to services (education, health care) for persons with disabilities.

In general, the results showed that the better the access to services, the higher the quality of life was for persons with disabilities; and that the greater the level of deprivation in the area (higher unemployment, higher share of persons receiving social benefits), the lower the individual quality of life. It is also to worth noting that the influence of access to services was stronger than that of the level of deprivation (for models without the size of the place of residence). The effects of the environmental factors (services and living conditions) were also much stronger than those of the LHDI.

## Discussion

In this research, we examined the intertwined relationship of three concepts: the first concept is disability and the limitations in functioning that disability causes; the second concept is a multidimensional quality of life; and the third concept is the environmental conditions created both for the entire population and for the population with disabilities. In addition, we examined a fourth dimension: namely, the locality expressed in measuring environmental factors at the local level. To our knowledge, no previous studies on particular countries or regions (provinces) or cross-country comparisons have dealt with all four of these aspects at the same time. There are, however, some existing studies that examined quality of life and environmental factors in various settings. For example, Celemin et al. [72] measured bivariate spatial autocorrelation between the Local Quality of Life Index and the HDI (Moran’s I = 0.412), and later disaggregated the global values to analyze local variations at the municipal level of the province of Buenos Aires and in the Autonomous City of Buenos Aires. Like in our study, the analysis was conducted at the local level, and was focused on linking the quality of life with the HDI. However, the authors used aggregated indices at the municipal level, whereas in our study, we matched individual QoL scores with the LHDI, and also introduced environmental variables representing the conditions created at the local level that were targeted at persons with disabilities. In addition, several previous studies have found that the external environment and its resources have a significant impact on the functioning of persons with disabilities, usually at the country level [69, 73, 74]. Those studies combined the concept of disability and the impact of environmental barriers (factors) on different life domains. Such barriers are associated with climate conditions, finances, and access to good-quality public services (including social services, but also transportation and spatial accessibility), the magnitude of which depends on individuals having more health problems, less physical independence, and poorer mental health. In another study conducted by Fellinghauer et al. [75], the authors broke down disability into difficulties in different components of functioning, such as impairments and limitations in activities and participation, and they examined the influence of these components on the QoL. Previous studies have reported the seemingly surprising result that persons with severe impairments tend to report a high quality of life [71, 76], including a high level of perceived health, regardless of their condition – this is the so-called “disability paradox.” Fellinghauer et al. [75] argued that contextual factors (i.e., the individual’s personal and environmental situation) play a crucial role in explaining this paradox. These results are in line with those of our study, as we also found that environmental factors influenced the quality of life. However, this research did not relate directly to the local level, and the quality of life was understood through one of its domains; i.e., either through activities and participation or through health.

Taking a broader approach, the results of our paper can also refer to other studies on the environmental barriers that the population with disabilities typically encounter. Many studies have shown that individuals with disabilities face more severe environmental barriers than individuals without disabilities [70, 77, 78]. There are also studies on the relationship between environmental conditions at the local level and health or daily functioning for the vulnerable population of older persons, who tend to experience limitations of daily living similar to those of the population with disabilities [79–82]. The findings of these studies highlight the important role the environment plays in different life domains.

The results of our research have some implications for the policy agenda aimed at persons with disabilities in Poland. First, it have provided crucial information that can be used in developing evidence-based policies targeted to the population with disabilities at the local level [83]. Special attention should be paid to the opportunities created in the local environment, while taking into account the different needs of persons with disabilities. Our results emphasize the necessity to create public policies aimed at persons with disabilities at the local level. This idea is quite obvious. For more details on the policy recommendations stemming from our analysis, we refer to the work of Toro-Hernandez et al. [84], in which the authors analyzed the factors that limit access to community assets for the population with disabilities. Taking this approach, we can see the connection between policies designed to facilitate general improvements in living conditions, and measures targeted at persons with disabilities. The policies aimed at persons with disabilities should be seek to improve their access to community assets, which are created for the entire population. Thus, public policies at the local level should be aimed not only at creating additional services or assets for the population with disabilities, but at making the existing ones available to the population with disabilities. Such policy measures should take into account the limitations faced by the population with disabilities at the personal level (e.g., lack of financial resources, inaccessible housing), interpersonal level (e.g., lack of personal assistance or aid), and community level (e.g., lack of accessible public transportation and inaccessible buildings) [84, 85]. The failure to implement measures that enable persons with disabilities to use community assets and to take advantage of other opportunities to enhance their life chances can be seen as discriminatory or exclusionary towards this population [1, 86].

Measures or regulations created at the state level seem to be insufficient, as in many cases the above-mentioned limitations are made worse by system-level barriers (e.g., a lack of effective enforcement of the legal framework). Thus, having a good understanding of the limitations of the living conditions of persons with disabilities is crucial. Such limitations should be identified at the local level in particular, as it is only by using this approach that measures can be tailored to the specific needs of the local population. One method that could be used to develop such measures is the service design process, which enables policymakers to look at solutions through the eyes of persons with different types of disabilities e.g., [87]. In practice, using this approach demands more personalization of support schemes for persons with disabilities, given that the extent to which persons with disabilities can achieve a good quality of life is influenced by the nature of their impairments; their individual, family, and community characteristics; as well as the environmental conditions created at local levels [88].

The conditions that enable persons with disabilities to enjoy a higher quality of life through the creation of high-quality living spaces are mostly shaped by local authorities. Hence, the institutional efficiency of local authorities, and their ability to listen to and cooperate with other crucial actors, are also essential elements in this context. Local policy should be shaped in constant dialogue with persons with disabilities and their representatives, care providers, service providers, and other institutions supporting persons with disabilities, especially non-governmental organizations [89]. The cooperation of these stakeholders can increase access to different assets and services for persons with disabilities, which can, in turn, increase their quality of life.

Local conditions and living space influence the overall multidimensional quality of life through their impacts on particular dimensions of quality of life. These impacts can be associated with access to high-quality services, especially social services. This is clearly visible in such dimensions as health or education [90]. Our research results confirm these findings, and draw attention to social services as a crucial determinant of the multidimensional quality of life for persons with disabilities.

Our research also highlights that material conditions, and opportunities to improve those conditions in the local areas where persons with disabilities live, are important determinants of multidimensional QoL. The main reasons why the population with disabilities tend to have poor material conditions it that their economic activities are limited and they need more medical treatment and rehabilitation than the general population. Allmark and Machaczek [91] reported similar results, and suggested that improving the financial capabilities of persons with disabilities would directly improve their QoL, and would indirectly improve their health. Although social benefits and allowances are regulated in Poland by state law, there are many possibilities at the local level to improve the financial situation of persons with disabilities, such as through the creation of job opportunities at social enterprises [92].

### Limitations

The basic limitation of our study is the scarcity of the data on livability and environmental conditions at the local level, especially in the context of disability. Thus, there is a need for a proper system that monitors these conditions, and fully monitors compliance with the UNCRPD. Having a broader set of livability indicators would diversify and enrich the analysis. Another potential subject for future qualitative and quantitative analysis is the expiration of the livability concept for persons with disabilities. A big opportunity for future research is to further develop the model for measuring QoL so that it can perform complex analyses of overall QoL, as well as of QoL in particular domains. The model can also be used for cross-country and cross-group comparisons.

## Conclusions

Our original contribution in this research is of both a methodological and a cognitive nature. First, based on the literature review in the conceptual part, we established a multidimensional model for measuring QoL, which can be applied not only to the population with disabilities (as we did in this paper), but also more broadly to other population groups, thereby enabling comparisons between groups. Second, we linked the concept of livability with the disability context. Moreover, we provided a measurement concept of livability, both for general living conditions and for conditions created with a specific focus on persons with disabilities. Finally, we used an analytical approach to measure the relationship between the individual quality of life of the population with disabilities and the external (environmental) conditions expressed by livability indicators at the LAU level 1 in Poland. This research design allowed us to quantify the meaning of external factors for the multidimensional quality of life of the population with disabilities. We demonstrated that environmental conditions played an important role in shaping the individual quality of life of persons with disabilities. Moreover, we found that the environmental conditions that had an impact on the QoL of this group were not just general conditions, but the conditions created especially for persons with disabilities. This illustrates the need to strengthen the policies aimed at persons with disabilities.

Moreover, we introduced the local level as a crucial issue in the creation of a disability-friendly environment. In our approach, we focused on the concept of livable local areas, which should create capabilities for persons with disabilities to achieve a higher QoL. Such an approach is in line with the UNCRPD, which highlighted the importance of the local environmental conditions in protecting the rights of persons with disabilities.

## Supplementary Information

**Additional file 1.** Annex 1

**Additional file 2.** Annex 2

## Data Availability

The datasets generated and/or analysed during the current study are available in the Eurostat repository, https://ec.europa.eu/eurostat/web/microdata/european-union-statistics-on-income-and-living-conditions

## Abbreviations

Deff: design effect
EU-SILC: European Union Statistics on Income and Living Conditions
ICC: interclass correlation coefficient
LAU: local administrative units
LHDI: Local Human Development Index
QoL: quality of life
MIMIC: multiple indicators multiple causes
UNCRPD: The United Nations Convention on the Rights of Persons with Disabilities
