# Supplementary material for: Individual quality of life and the environment – towards a concept of livable areas for persons with disabilities in Poland
Source: BMC Public Health. 2021 Apr 17;21:740. doi: 10.1186/s12889-021-10797-7 (PMC8052840; doi:10.1186/s12889-021-10797-7)
Supplement: Supplementary file 1 — Additional file 1. Annex 1 [file 12889_2021_10797_MOESM1_ESM.docx]

**Individual Quality of Life and the Environment – Towards a Concept of Liveable Areas for Persons with Disabilities in Poland**

Izabela Grabowska*, PhD, igrabow@sgh.waw.pl, SGH Warsaw School of Economics, Institute of Statistics and Demography, Warsaw, Poland – corresponding author, IG

Radosław Antczak, PhD, rantcza@sgh.waw.pl, SGH Warsaw School of Economics, Institute of Statistics and Demography, Warsaw, Poland, RA

Jan Zwierzchowski, PhD, jzwier@sgh.waw.pl, SGH Warsaw School of Economics, Institute of Statistics and Demography, Warsaw, Poland, JZ

Tomasz Panek, Professor, tompa10@interia.pl, SGH Warsaw School of Economics, Institute of Statistics and Demography, Warsaw, Poland, TP

**Annex 1. List of variables used as symptoms for each life quality domain**

| **Domains** | **Indicators** |
| --- | --- |
| 1. Material conditions | 1.1. Median disposable equivalised income  1.2. At-risk-of poverty rate  1.3. At-risk-of poverty rate anchored at a fixed moment in time  1.4. Satisfaction with financial situation  1.5. Severe material deprivation rate  1.6. (In) ability to make ends meet  1.7. Structural problems of the dwelling  1.8. Space of dwelling overcrowding/under-occupation |
| 2. Productivity or other main activity  (Productivity) | 2.1. Employment rate  2.2. Unemployment rate  2.3. Long-term unemployment rate  2.4. People living in households with very low work intensity  2.5. Underemployed part-time workers  2.6. Low-wage earners  2.7. Long working hours (more than 48 at week)  2.8. Job satisfaction |
| 3. Health | 3.1. Self-perceived health  3.2. Unmet needs for medical care |
| 4. Education | 4.1. Educational attainment  4. 2. Early leavers from education and training |
| 5. Leisure and social interactions  (Leisure-Social-Interactions) | 5.1. Non-participation in culture or sport activities  5. 2. Satisfaction with time use  5. 3. Financial obstacles to leisure participation  5.4. Frequency of getting together with friends  5.5. Satisfaction with personal relationships  5.6. Participation in voluntary activities  5.7. Help from others (having someone to rely on in case of need)  5.8 Having someone to discuss personal matters with  5.9 Trust in others |
| 6. Economic security and physical safety  (Security-Safety) | 6.1. Population unable to face unexpected financial expenses  6.2. Population in arrears  6.3. Perception of crime, violence, and vandalism in the living area  6.4. Feeling of safety (people feeling safe when walking alone in their area after dark) |
| 7. Governance and basic rights  (Basic-Rights) | 7.1. Trust in the Parliament, the local authorities, the government, the courts, the police  7.2. Active citizenship |
| 8. Natural and living environment  (Environment) | 8.1. Perception of pollution, grime, and other environmental problems  8.2. Noise from neighbours or from the street  8.3. Satisfaction with recreational and green areas  8.4. Satisfaction with living environment |
| 9. Overall experience of life  (Experience-Life) | 9.1. Overall life satisfaction  9.2. Negative affect (being very nervous, feeling down in the dumps, feeling downhearted or depressed)  9.3. Positive affect (being happy, being calm, feeling full of life)  9.4. Assessing whether life is worthwhile |
