# Supplementary material for: Individual quality of life and the environment – towards a concept of livable areas for persons with disabilities in Poland
Source: BMC Public Health. 2021 Apr 17;21:740. doi: 10.1186/s12889-021-10797-7 (PMC8052840; doi:10.1186/s12889-021-10797-7)
Supplement: Supplementary file 2 — Additional file 2. Annex 2 [file 12889_2021_10797_MOESM2_ESM.docx]

**Individual Quality of Life and the Environment – Towards a Concept of Liveable Areas for Persons with Disabilities in Poland**

Izabela Grabowska*, PhD, igrabow@sgh.waw.pl, SGH Warsaw School of Economics, Institute of Statistics and Demography, Warsaw, Poland – corresponding author, IG

Radosław Antczak, PhD, rantcza@sgh.waw.pl, SGH Warsaw School of Economics, Institute of Statistics and Demography, Warsaw, Poland, RA

Jan Zwierzchowski, PhD, jzwier@sgh.waw.pl, SGH Warsaw School of Economics, Institute of Statistics and Demography, Warsaw, Poland, JZ

Tomasz Panek, Professor, tompa10@interia.pl, SGH Warsaw School of Economics, Institute of Statistics and Demography, Warsaw, Poland, TP

**Annex 2. Results of the MIMIC model for all QoL dimensions**

| **Material conditions** | | | | | | |
| --- | --- | --- | --- | --- | --- | --- |
| Structural part |  | **Variable** | **Coefficient** | **Std. Err.** | **z** | **P>\|z\|** |
|  |  | Productivity | 0.42193 | 0.70315 | 0.600057 | 0.548468 |
|  |  | Health | -0.10018 | 0.82252 | -0.1218 | 0.90306 |
|  |  | Education | 0.3032 | 0.49733 | 0.609656 | 0.54209 |
|  |  | Age | 0.20089 | 0.20182 | 0.995392 | 0.319546 |
|  |  | Household size | -0.28209 | 0.28335 | -0.99555 | 0.319467 |
| Measurement part | **Equation** | **Variable** | **Coefficient** | **Std.Err.** | **z** | **P>\|z\|** |
|  | Median disposable equivalised income | Material_conditions | 0.66373 | 0.26045 | 2.548397 | 0.010822 |
|  |  | Constant term | 1.51087 | 0.92292 | 1.637054 | 0.101619 |
|  | At-risk-of poverty rate | Material_conditions | 0.76554 | 0.13006 | 5.886053 | 3.96E-09 |
|  |  | Constant term | 0.16455 | 0.57027 | 0.288548 | 0.772928 |
|  | At-risk-of poverty rate anchored at a fixed moment in time | Material_conditions | 0.60835 | 0.2108 | 2.885911 | 0.003903 |
|  |  | Constant term | 0.33382 | 0.722 | 0.462355 | 0.643827 |
|  | Satisfaction with financial situation | Material_conditions | 0.60394 | 0.19412 | 3.111168 | 0.001863 |
|  |  | Constant term | 0.29686 | 0.55939 | 0.530685 | 0.595637 |
|  | Severe material deprivation rate | Material_conditions | 0.87951 | 0.81915 | 1.073686 | 0.282963 |
|  |  | Constant term | 0.5425 | 0.37834 | 1.433895 | 0.151602 |
|  | (In) ability to make ends meet | Material_conditions | -0.81455 | 0.53342 | -1.52703 | 0.126753 |
|  |  | Constant term | 1.87939 | 0.24383 | 7.707788 | 1.29E-14 |
|  | Structural problems of the dwelling | Material_conditions | 0.58228 | 0.81562 | 0.713911 | 0.475282 |
|  | Structural problems of the dwelling | Constant term | 0.28574 | 0.08253 | 3.462256 | 0.000536 |
|  | Space of dwelling overcrowding/under-occupation | Material_conditions | 0.39416 | 0.23506 | 1.676848 | 0.093572 |
|  | Space of dwelling overcrowding/under-occupation | Constant term | 0.77324 | 0.76129 | 1.015697 | 0.309774 |
| **Productivity** | | | | | | |
| Structural part |  | **Variable** | **Coefficient** | **Std. Err.** | **z** | **P>\|z\|** |
|  |  | Health | -0.14773 | 0.3706 | -0.39862 | 0.69017 |
|  |  | Education | -0.11729 | 0.00596 | -19.6795 | 3.23E-86 |
|  |  | Sex | 0.02258 | 0.15244 | 0.148124 | 0.882245 |
|  |  | Student | -0.44483 | 0.29382 | -1.51395 | 0.130037 |
|  |  | Age | -0.05734 | 0.02385 | -2.40419 | 0.016208 |
|  |  | Big city | 0.01358 | 0.00585 | 2.321368 | 0.020267 |
|  |  | Medium town | 0.02202 | 0.03108 | 0.708494 | 0.478638 |
|  |  | Small town | 0.0228 | 0.0455 | 0.501099 | 0.616302 |
|  |  | Rural | -0.01153 | 0.10231 | -0.1127 | 0.910271 |
|  |  | Health limitations | 0.16718 | 0.31809 | 0.525575 | 0.599184 |
|  |  | Penioner | -0.68495 | 0.27161 | -2.52181 | 0.011675 |
|  |  | Disability pensioner | -0.32721 | 0.4021 | -0.81375 | 0.415787 |
|  |  | Household size | 0.00338 | 0.37962 | 0.008904 | 0.992896 |
|  |  | Presence of the partner | 0.0873 | 0.33411 | 0.261291 | 0.793868 |
|  |  | Unemployed | -0.38763 | 0.85907 | -0.45122 | 0.651831 |
|  |  | Inactive | -0.42016 | 0.13864 | -3.03058 | 0.002441 |
| Measurment part | **Equation** | **Variable** | **Coefficient** | **Std. Err.** | **z** | **P>\|z\|** |
|  | Employment rate | Productivity | 0.89025 | 0.14051 | 6.335848 | 2.36E-10 |
|  |  | Constant term | 0.73264 | 0.09934 | 7.375075 | 1.64E-13 |
|  | Unemployment rate | Productivity | -0.29124 | 0.8398 | -0.3468 | 0.728744 |
|  |  | Constant term | 0.20025 | 0.51302 | 0.390336 | 0.696288 |
|  | Long-term unemployment rate | Productivity | -0.23384 | 0.66232 | -0.35306 | 0.724042 |
|  |  | Constant term | 0.16333 | 0.21712 | 0.752257 | 0.451897 |
|  | People living in households with very low work intensity | Productivity | 0 | 0 | 0.01 | 0.992021 |
|  |  | Constant term | 0.000099 | 0.000041 | 2.414634 | 0.015751 |
|  | Underemployed part-time workers | Productivity | 0.1863 | 0.39375 | 0.473143 | 0.636111 |
|  |  | Constant term | 0.16361 | 0.91417 | 0.178971 | 0.85796 |
|  | Low-wage earners | Productivity | 0.51634 | 0.42894 | 1.203758 | 0.228683 |
|  |  | Constant term | 0.52894 | 0.51063 | 1.035858 | 0.300269 |
|  | Long working hours (more than 48 at week) | Productivity | -0.10875 | 0.03787 | -2.87167 | 0.004083 |
|  |  | Constant term | 0.87345 | 0.74687 | 1.169481 | 0.24221 |
|  | Job satisfaction | Productivity | 0.90188 | 0.14505 | 6.217718 | 5.04E-10 |
|  |  | Constant term | 0.89963 | 0.7849 | 1.146171 | 0.251724 |
| **Health** | | | | | | |
| Structural part |  | **Variable** | **Coefficient** | **Std.Err.** | **z** | **P>\|z\|** |
|  |  | Education | -0.25852 | 0.64496 | -0.40083 | 0.688545 |
|  |  | Sex | 0.03902 | 0.02342 | 1.666097 | 0.095694 |
|  |  | Age | -0.23201 | 0.18302 | -1.26768 | 0.204914 |
|  |  | Big city | 0.01681 | 0.34063 | 0.04935 | 0.960641 |
|  |  | Medium town | 0.04734 | 0.13189 | 0.358935 | 0.719643 |
|  |  | Small town | 0.05612 | 0.33566 | 0.167193 | 0.867218 |
|  |  | Rural | 0.03893 | 0.55162 | 0.070574 | 0.943737 |
|  |  | Pensioner | 0.06179 | 0.25101 | 0.246165 | 0.805554 |
|  |  | Disability pensioner | 0.34959 | 0.42589 | 0.820846 | 0.411734 |
| Measurement part | **Equation** | **Variable** | **Coefficient** | **Std.Err.** | **z** | **P>\|z\|** |
|  | Self-perceived health | Health | -0.88057 | 0.4016 | -2.19265 | 0.028332 |
|  |  | Constant term | 0.8172 | 0.50836 | 1.607522 | 0.10794 |
|  | Unmet needs for medical care | Health | -0.01992 | 0.97402 | -0.02045 | 0.983683 |
|  |  | Constant term | 0.39856 | 0.15817 | 2.51982 | 0.011741 |
| **Education** | | | | | | |
| Structural part |  | **Variable** | **Coefficient** | **Std.Err.** | **z** | **P>\|z\|** |
|  |  | Sex | 0.01445 | 0.0806 | 0.17928 | 0.857718 |
|  |  | Student | -0.21624 | 0.14688 | -1.47222 | 0.140961 |
|  |  | Age | -0.0538 | 0.51436 | -0.1046 | 0.916696 |
|  |  | Big city | -0.04439 | 0.57715 | -0.07691 | 0.938693 |
|  |  | Medium town | -0.05482 | 0.4957 | -0.11059 | 0.911941 |
|  |  | Small town | -0.18019 | 0.90656 | -0.19876 | 0.842449 |
|  |  | Rural | -0.32235 | 0.85965 | -0.37498 | 0.707677 |
|  |  | Household size | 0.15654 | 0.19676 | 0.795589 | 0.426271 |
| Measurement part | **Equation** | **Variable** | **Coefficient** | **Std.Err.** | **z** | **P>\|z\|** |
|  | Educational attainment | Education | 0.7107 | 0.24015 | 2.9594 | 0.003082 |
|  |  | Constant term | 0.3266 | 0.8024 | 0.407029 | 0.683987 |
|  | Early leavers from education and training | Education | -0.84177 | 0.199 | -4.23 | 2.34E-05 |
|  |  | Constant term | 0.24432 | 0.02181 | 11.2022 | 0 |
| **Leisure and social interactions** | | | | | | |
| Structural part |  | **Variable** | **Coefficient** | **Std.Err.** | **z** | **P>\|z\|** |
|  |  | Material_conditions | 0.32095 | 0.01049 | 30.59581 | 0 |
|  |  | Productivity | -1.83185 | 0.96176 | -1.90469 | 0.056821 |
|  |  | Pensioner | -1.30323 | 0.34784 | -3.74664 | 0.000179 |
|  |  | Presence of the partner | 0.25878 | 0.04609 | 5.614667 | 1.97E-08 |
|  |  | Inactive | -0.71612 | 0.34644 | -2.06708 | 0.038726 |
| Measurement part | **Equation** | **Variable** | **Coefficient** | **Std.Err.** | **z** | **P>\|z\|** |
|  | Non-participation in culture or sport activities | Leisure_Social_Interactions | 0.93655 | 0.15554 | 6.021281 | 1.73E-09 |
|  |  | Constant term | 0.20274 | 0.20352 | 0.996167 | 0.319169 |
|  | Satisfaction with time use | Leisure_Social_Interactions - | -0.27367 | 0.02343 | -11.6803 | 1.61E-31 |
|  |  | Constant term | 0.15222 | 0.14505 | 1.049431 | 0.29398 |
|  | Financial obstacles to leisure participation | Leisure_Social_Interactions | 0.73015 | 0.62372 | 1.170637 | 0.241745 |
|  |  | Constant term | 0.23602 | 0.20815 | 1.133894 | 0.256839 |
|  | Frequency of getting together with friends | Leisure_Social_Interactions - | -0.9232 | 0.15955 | -5.78627 | 7.2E-09 |
|  |  | Constant term | 0.05621 | 0.05346 | 1.05144 | 0.293056 |
|  | Satisfaction with personal relationships | Leisure_Social_Interactions | 0.65663 | 0.35678 | 1.840434 | 0.065705 |
|  |  | Constant term | 0.6297 | 0.27457 | 2.293404 | 0.021825 |
|  | Participation in voluntary activities | Leisure_Social_Interactions | 0.99338 | 0.01111 | 89.41314 | 0 |
|  |  | Constant term | 0.52595 | 0.60477 | 0.869669 | 0.384481 |
|  | Help from others (having someone to rely on in case of need) | Leisure_Social_Interactions | 0.9921 | 0.01853 | 53.54021 | 0 |
|  |  | Constant term | 0.52456 | 0.61594 | 0.851641 | 0.394413 |
|  | Trust in others | Leisure_Social_Interactions - | -0.58101 | 0.45244 | -1.28417 | 0.199082 |
|  |  | Constant term | 0.30391 | 0.48545 | 0.626038 | 0.53129 |
| **Economic security and physical safety** | | | | | | |
| Structural part |  | **Variable** | **Coefficient** | **Std.Err.** | **z** | **P>\|z\|** |
|  |  | Material_conditions | -0.89705 | 0.62628 | -1.43235 | 0.152045 |
|  |  | Sex | -0.02116 | 0.61728 | -0.03428 | 0.972654 |
|  |  | Age | 0.14684 | 0.50394 | 0.291384 | 0.770758 |
|  |  | Big city | -0.04141 | 0.44144 | -0.09381 | 0.925263 |
|  |  | medium town | -0.04426 | 0.24854 | -0.17808 | 0.85866 |
|  |  | Small town | -0.11189 | 0.91877 | -0.12178 | 0.903071 |
|  |  | Rural | -0.29155 | 0.44492 | -0.65529 | 0.512283 |
|  |  | Presence of the partner | -0.01292 | 0.96981 | -0.01332 | 0.989371 |
| Measurment part | **Equation** | **Variable** | **Coefficient** | **Std.Err.** | **z** | **P>\|z\|** |
|  | Perception of crime. violence. and vandalism in the living area | Security_safety | -0.78587 | 0.4893 | -1.60611 | 0.10825 |
|  |  | Constant term | 0.68511 | 0.09687 | 7.072468 | 1.52E-12 |
|  | Population unable to face unexpected financial expenses | Security_safety | 0.3465 | 0.41871 | 0.827542 | 0.40793 |
|  |  | Constant term | 0.29515 | 0.04772 | 6.185038 | 6.21E-10 |
|  | Feeling of safety (people feeling safe when walking alone in their area after dark) | Security_safety | 0.07096 | 0.50923 | 0.139348 | 0.889175 |
|  |  | Constant term | 0.24543 | 0.08928 | 2.748992 | 0.005978 |
|  | Population in arrears | Security_safety | -0.03227 | 0.03521 | -0.9165 | 0.359404 |
|  |  | Constant term | 0.13185 | 0.01525 | 8.645902 | 0 |
| **Governance and basic rights** | | | | | | |
| Structural part |  | **Variable** | **Coefficient** | **Std.Err.** | **z** | **P>\|z\|** |
|  |  | Sex | -0.16975 | 0.0214 | -7.93224 | 2.15E-15 |
|  |  | Age | -0.08221 | 0.02352 | -3.49532 | 0.000473 |
|  |  | Big city | 0.03153 | 0.03306 | 0.953721 | 0.340225 |
|  |  | Medium town | 0.04065 | 0.03423 | 1.187555 | 0.235009 |
|  |  | Small town | 0.04944 | 0.05719 | 0.864487 | 0.387321 |
|  |  | Rural | 0.06889 | 0.05191 | 1.327105 | 0.184474 |
| Measurement part | **Equation** | **Variable** | **Coefficient** | **Std.Err.** | **z** | **P>\|z\|** |
|  | Trust in the Parliament | Basic_rights | 2.476419 | 0.03734 | 66.32 | 0 |
|  |  | Constant term | 3.148847 | 0.038001 | 82.86 | 0 |
|  | Trust in the local authorities | Basic_rights | 1.230928 | 0.022863 | 53.84 | 0 |
|  |  | Constant term | 2.551202 | 0.019373 | 131.69 | 0 |
|  | Trust in the government | Basic_rights | 2.524901 | 0.038049 | 66.36 | 0 |
|  |  | Constant term | 3.136462 | 0.038734 | 80.97 | 0 |
|  | Trust in courts | Basic_rights | 1.303944 | 0.023738 | 54.93 | 0 |
|  |  | Constant term | 2.606444 | 0.020462 | 127.38 | 0 |
|  | Trust in the police | Basic_rights | 1 | (constrained) |  |  |
|  |  | Constant term | 2.394372 | 0.015991 | 149.74 | 0 |
|  | Active citizenship | Basic_rights | 0.0193287 | 0.003826 | 5.05 | 0 |
|  |  | Constant term | 0.0526302 | 0.001138 | 46.27 | 0 |
| **Natural and living enviroment** | | | | | | |
| Structural part |  | **Variable** | **Coefficient** | **Std.Err.** | **z** | **P>\|z\|** |
|  |  | Big city | 0.14631 | 0.0591 | 2.475635 | 0.0133 |
|  |  | Medium town | 0.40195 | 0.159 | 2.527987 | 0.011472 |
|  |  | Small town | -0.37712 | 0.16562 | -2.27702 | 0.022785 |
|  |  | Rural | -0.9258 | 0.38158 | -2.42623 | 0.015257 |
|  |  | Household size | 0.32967 | 0.12842 | 2.567124 | 0.010255 |
| Measurement part | **Equation** | **Variable** | **Coefficient** | **Std.Err.** | **z** | **P>\|z\|** |
|  | Perception of pollution. grime. and other environmental problems | Environment | -0.27288 | 0.13162 | -2.07324 | 0.03815 |
|  |  | Constant term | 0.29156 | 0.02245 | 12.98708 | 0 |
|  | Perception of pollution. grime. and other environmental problems | Environment | 0.22551 | 0.10934 | 2.062466 | 0.039163 |
|  |  | Constant term | 0.39385 | 0.02236 | 17.61404 | 0 |
|  | Satisfaction with recreational and green areas | Environment | 0.00 | 0.00 | -0.00068 | 0.999455 |
|  |  | Constant term | 0.00001 | 0.000054 | 0.185185 | 0.853084 |
|  | Satisfaction with living environment | Environment | -0.07891 | 0.03584 | -2.20173 | 0.027684 |
|  |  | Constant term | 0.29369 | 0.04691 | 6.260712 | 3.83E-10 |
| **Overall experience of life (subjective wellbeing)** | | | | | | |
| Structural part |  | **Variable** | **Coefficient** | **Std.Err.** | **z** | **P>\|z\|** |
|  |  | Student | 0.0390479 | 0.0055 | 7.1 | 0 |
|  |  | Age | -0.0383985 | 0.007949 | -4.83 | 0 |
|  |  | Gender | -0.0159984 | 0.004506 | -3.55 | 0 |
|  |  | Big city | -0.0319172 | 0.005565 | -5.74 | 0 |
|  |  | Medium town | -0.0387427 | 0.00475 | -8.16 | 0 |
|  |  | Self perceived health | 0.2443572 | 0.006207 | 39.37 | 0 |
|  |  | Monetary_poor | -0.0245198 | 0.00474 | -5.17 | 0 |
|  |  | Material deprivation | -0.1661296 | 0.005357 | -31.01 | 0 |
|  |  | Unemployed | -0.0398439 | 0.004384 | -9.09 | 0 |
|  |  | Retired | 0.0383867 | 0.005485 | 7 | 0 |
|  |  | Income | 0.0204196 | 0.005522 | 3.7 | 0 |
|  |  | Unmet_medical_needs | -0.0439949 | 0.004214 | -10.44 | 0 |
|  |  | Arrears | -0.0461686 | 0.004845 | -9.53 | 0 |
|  |  | Crime | -0.0131231 | 0.004733 | -2.77 | 0.006 |
|  |  | Household size | 0.0173091 | 0.005489 | 3.15 | 0.002 |
| Measurement part | **Equation** | **Variable** | **Coefficient** | **Std.Err.** | **z** | **P>\|z\|** |
|  | Overall life satisfaction | Subjective_wellbeing | 1 | (constrained) |  |  |
|  |  | Constant term | 0.0283563 | 0.006953 | 4.08 | 0 |
|  | Assessing whether life is worthwhile | Subjective_wellbeing | 0.5287069 | 0.012749 | 41.47 | 0 |
|  |  | Constant term | 0.0292289 | 0.007641 | 3.83 | 0 |
|  | Positive affect - full of life | Subjective_wellbeing | 1.093063 | 0.013082 | 83.55 | 0 |
|  |  | Constant term | 0.0224684 | 0.006933 | 3.24 | 0.001 |
|  | Positive affect - calm | Subjective_wellbeing | 0.7752744 | 0.012606 | 61.5 | 0 |
|  |  | Constant term | 0.0160439 | 0.007068 | 2.27 | 0.023 |
|  | Positive affect - happy | Subjective_wellbeing | 1.075263 | 0.013023 | 82.56 | 0 |
|  |  | Constant term | 0.0280781 | 0.006911 | 4.06 | 0 |
|  | Negative affect - depressed | Subjective_wellbeing | -1.00162 | 0.013243 | -75.64 | 0 |
|  |  | Constant term | -0.0317269 | 0.006912 | -4.59 | 0 |
|  | Negative affect - nervous | Subjective_wellbeing | -0.764376 | 0.012782 | -59.8 | 0 |
|  |  | Constant term | -0.031396 | 0.007049 | -4.45 | 0 |
|  | Negative affect - sad | Subjective_wellbeing | -1.018863 | 0.01335 | -76.32 | 0 |
|  |  | Constant term | -0.0301737 | 0.006929 | -4.35 | 0 |

Source: own study.
